# A MOSAIC OF RESILIENCE: INDIVIDUAL AND SOCIAL FACTORS FOR AGING WELL AMONG DIVERSE OLDER ADULTS

**DOI:** 10.1093/geroni/igae098.1140

**Published:** 2024-12-31

**Authors:** Aisha Cozad, Cassandra Cantave Burton, Fanni Farago

**Affiliations:** AARP, Washington, District of Columbia, United States; AARP, Washington, District of Columbia, United States; AARP, District of Columbia, District of Columbia, United States

## Abstract

In the wake of the COVID-19 pandemic and continuing extreme weather events, the resilience of older adults in the areas of health and disaster management and preparedness are critical areas of study. As shown by AARP survey research, self-reported happiness and emotional well-being and resiliency is high among older adults, including across racial and ethnic groups. At the same time, these studies also demonstrate that many older adults have trouble bouncing back from hardships and feel concerned about the possibility of declining health and their ability to live independently. In related AARP research, survey data illuminates the crucial role that quality relationships and community features, including affordable and accessible housing, healthcare, and opportunities for social engagement, play in providing structural supports for older adults to age well in their homes and communities. Drawing on multiple AARP survey studies, this presentation will explore different aspects of resilience in aging among diverse communities of older adults, including those who identify as Black/African American, Hispanic/Latino, or LGBTQ.

